# Tumor-associated macrophage-derived GDNF promotes gastric cancer liver metastasis via a GFRA1-modulated autophagy flux

**DOI:** 10.1007/s13402-022-00751-z

**Published:** 2023-02-20

**Authors:** Bo Ni, Xuan He, Yeqian Zhang, Zeyu Wang, Zhongyi Dong, Xiang Xia, Gang Zhao, Hui Cao, Chunchao Zhu, Qing Li, Jiahua Liu, Huimin Chen, Zizhen Zhang

**Affiliations:** 1 Department of Gastrointestinal Surgery, Renji Hospital, Shanghai Jiao Tong University School of Medicine, 160 Pujian Road, Shanghai, China; 2 State Key Laboratory of Oncogenes and Related Genes, Shanghai Cancer Institute, Renji Hospital, Shanghai Jiao Tong University School of Medicine, Shanghai, China; 3 Department of Pharmacy, Ruijin Hospital, Shanghai Jiao Tong University School of Medicine, Shanghai, China; 4 State Key Laboratory for Oncogenes and Related GenesKey Laboratory of Gastroenterology & Hepatology, Ministry of HealthDivision of Gastroenterology and HepatologyShanghai Institute of Digestive DiseaseRenji Hospital, Shanghai Jiao Tong University School of Medicine, Shanghai, China

**Keywords:** Gastric cancer, Liver metastasis, Tumor associated macrophage, Autophagy, GFRA1.

## Abstract

**Purpose:**

Liver metastasis, a lethal malignancy of gastric cancer (GC) patients, execrably impairs their prognosis. As yet, however, few studies have been designed to identify the driving molecules during its formation, except screening evidence pausing before their functions or mechanisms. Here, we aimed to survey a key driving event within the invasive margin of liver metastases.

**Methods:**

A metastatic GC tissue microarray was used for exploring malignant events during liver-metastasis formation, followed by assessing the expression patterns of glial cell-derived neurotrophic factor (GDNF) and GDNF family receptor alpha 1 (GFRA1). Their oncogenic functions were determined by both loss- and gain-of-function studies in vitro and in vivo, and validated by rescue experiments. Multiple cell biological studies were performed to identify the underlying mechanisms.

**Results:**

In the invasive margin, GFRA1 was identified as a pivotal molecule involved in cellular survival during liver metastasis formation, and we found that its oncogenic role depends on tumor associated macrophage (TAM)-derived GDNF. In addition, we found that the GDNF-GFRA1 axis protects tumor cells from apoptosis under metabolic stress via regulating lysosomal functions and autophagy flux, and participates in the regulation of cytosolic calcium ion signalling in a RET-independent and non-canonical way.

**Conclusion:**

From our data we conclude that TAMs, homing around metastatic nests, induce the autophagy flux of GC cells and promote the development of liver metastasis via GDNF-GFRA1 signalling. This is expected to improve the comprehension of metastatic pathogenesis and to provide a novel direction of research and translational strategies for the treatment of metastatic GC patients.

**Supplementary Information:**

The online version contains supplementary material available at 10.1007/s13402-022-00751-z.

## Introduction

With continuous improvements in diagnostic and treatment technologies and a continuous accumulation of evidence-based medicine, standardized surgery and comprehensive treatment options for gastric cancer (GC) some progress has been made in the past decades [1, 2]. As yet, however, the overall prognosis for this condition is still poor, and the age-standardized 5-year survival rate is only 27.4% [3, 4]. An explanation for this phenomenon is that distant metastasis caused by the high invasiveness of GC leads to a poor patient prognosis [5]. Due to its anatomic characteristics and venous return, the liver is one of the most common organs exhibiting metastasis [6]. A significant number of GC patients already has synchronous liver metastases at the time of diagnosis and, therefore, even aggressive comprehensive treatment fails to improve the very poor prognosis of such patients, resulting in a 5-year survival rate of less than 10%. Therefore, it is highly relevant to explore the mechanisms underlying GC liver metastasis [7, 8].

Liver metastasis of digestive system tumors is an extremely complex process that involves a series of multidimensional and multi-spatiotemporal regulatory processes, such as the infiltration and migration of primary tumor cells and the rooting and growth of circulating tumor cells [9–11]. The liver may provide a “soil” that affects the fate of tumor cells (also known as special tumor microenvironment (TME), particularly in the tumor invasive margin (IM) of liver tissue), is essential for the formation of metastatic niches [12–14]. In this process, tumor-associated macrophages (TAMs) may play an important role [15]. Some studies have proposed specific mechanisms by which TAMs may promote metastasis in breast cancer, pancreatic cancer and other diseases [16]. As yet, however, research on the role of TAMs in promoting GC metastasis has been sparce.

A genome-wide DNA methylation sequencing study has shown that the methylation level of the GDNF family receptor alpha 1 (GFRA1) promoter region is significantly reduced in GC patients with liver metastasis, suggesting that GFRA1 expression may be closely related to GC liver metastasis [17]. GFRA1 acts as a glycosylphosphatidylinositol (GPI)-linked receptor, and its ligands include glial cell-derived neurotrophic factor (GDNF) [18]. Due to lack of the intracellular segment of signal transduction, the classical signal transduction pathway mediated by GFRA1 relies on RET receptor tyrosine kinase to activate downstream signalling pathways [19, 20]. The GDNF-GFRA1 signalling axis has been found to be involved in the malignant progression of a variety of cancers, including pancreatic cancer, breast cancer and osteosarcoma, whereas its role and mechanism in GC liver metastasis has not been studied [21, 22].

Currently, increasing attention is being paid to the key steps involved in the process of tumor metastasis, by which circulating tumor cells (CSC) colonize and survive in target organs [23, 24]. After reaching its target organ, tumor cells need to overcome a harsh and unfamiliar growth environment in order to survive and colonize, and to proliferate to form active metastatic lesions [25, 26]. Autophagy is an important protective mechanism for tumor cells to resist harsh living environments, such as hypoxia and nutritional deprivation and, therefore, its relationship with distant metastasis warrants an in-depth investigation [27–29]. Here, we show that GFRA1 upregulation is closely related to GC liver metastasis. Both in vitro and in vivo experiments revealed that TAMs may enhance the autophagy level in GC cells with a positive GFRA1 expression by secreting GDNF, thereby helping TAMs to colonize and survive in metastatic niches. Our study may provide new potential targets for the treatment of metastatic GC.

## Materials and methods

### Human tissue samples

All specimens in this study were collected from the Department of Gastrointestinal Surgery, Renji Hospital. In total 69 samples from GC patients with liver metastasis were used to construct a metastatic tumor tissue microarray (TMA). Next to these, 20 fresh frozen tissue samples from GC patients were collected for RNA and protein extraction, half of which were single GC cases and the other half primary tumors and metastatic liver lesions. All participants provided informed consent under a Renji Hospital Ethics Committee approved protocol. The ethical approval number was (2017)114.

### Cells and culture conditions

Human GC cell lines AGS, HGC27, BGC823, MKN45, MKN28, MGC803 and a normal gastric mucosal epithelial cell line, GES-1, were preserved at the Shanghai Cancer Institute, while the mouse GC cell line MFC was purchased from the Cell Bank of Chinese Academy of Sciences (China). In addition, the THP-1 cell line was purchased from the American Type Culture Collection (USA). Cells were maintained in RPMI-1640 medium, supplemented with 10% heat-inactivated fetal bovine serum (FBS, 16,000,044, Gibco) and a 1% antibiotic mixture of penicillin/streptomycin (10,000 U/ml,15,140–122, Gibco), in an incubator at 37 °C and 5% CO_2_. Recombinant protein rGDNF (RP-8602, Thermo Fisher Scientific, USA), agonist BT18 (HY-111969, MCE, China), BT13 (HY-124401, MCE, China) and inhibitor bafilomycin A1 (HY-100558, MCE) were used in this study. Bafilomycin A1 was added to the culture medium 2 h before rGDNF application.

### Mouse experiments

Male BALB/C nude mice and C57BL/6 N mice (6–8 weeks old) were selected as in vivo models and fed according to the guidelines for the Care and Use of Laboratory Animals prepared by the National Academy of Sciences. All mice experimental procedures were approved by the Research Ethics Committee of Renji Hospital. Liver-metastasis and chemically depleted macrophage models were applied as outlined below.

### Liver metastasis model

Three groups of metastatic models were designed for different experimental purposes. Firstly, male BALB/C nude mice were anesthetized using 2.5% isoflurane (CHLCR00690790121, Avantor, China) after which 1 × 10^6^ AGS cells in 0.02 ml PBS, stably transduced with sh*GFRA1*-lentiviral vector, were injected into the spleen by insulin syringes to form in vivo liver metastasis. The formation of metastatic lesions took around 30 days. Secondly, male C57BL/6 N mice were pre-treated with PBS liposomes and clodronate liposomes, and next intra-splenic injected with 1 × 10^6^ ov-*GFRA1* MFC-luciferase cells two weeks later. The third group of mice was injected with MFC cells and treated with 1 mg/kg bafilomycin A1 and equivalent vehicles. These mice were maintained for luminescence detection and survival analysis, after which their livers were collected for further analysis (n = 8, respectively).

### Chemically depleted macrophage model

In order to eliminate bone marrow-derived macrophages, C57BL/6 N male mice were treated with 1.5 mg/20 g clodronate liposomes and the same volume of PBS liposomes twice a week by intraperitoneal injection. Clodronate liposomes and PBS liposomes were acquired from Dr. Nico van Rooijen (Vrije Universiteit, Netherlands). This reagent was applied two weeks before intrasplenic injection of MFC cells.

### Induction of tumor associated macrophages

Human THP-1 cells were stimulated with 100 ng/ml phorbol 12-myristate 13-acetate (PMA, Sigma, USA) to differentiate them into adherent Mφ macrophages. Next, Mφ macrophages were incubated with 40 ng/ml IL-4 (200–04, Peprotech, USA) and 20 ng/ml IL-13 (200–13, Peprotech, USA) to differentiate them into tumor associated macrophages.

### In vitro genome-editing

Lentivirus carrying shRNA plasmids against *GFRA1* and scrambled sequences were purchased from Genomeditech (China) for obtaining stable *GFRA1* knockdown in AGS and HGC27 cells. 1 × 10^8^ units of lentivirus were transduced into 1 × 10^6^ GC cells with 10 μg/ml polybrene (H9268, Sigma-Aldrich) transfection reagent. 24 h later, transfected cells were incubated with 2 μg/ml puromycin (A1113802, Gibco, USA) for three days to obtain gene-edited cells.

For transient interference of *GDNF*, siRNAs were synthesized by Genomeditech, and transfected into TAMs with Lipofectamine® RNAiMAX (13,778,150, Invitrogen, USA). The interference sequences used were as follows:

**Table:** 

*GFRA1*-sh1	5’-GGGAGAAGCCCAACUGUUUTT-3’
*GFRA1*-sh2	5’- CCUAGAAGAGUGCUUGAAATT -3’
GDNF	5’-GCCAGTGTTTATCTGATAC-3’

Lentiviruses carrying full-length cDNA encoding mouse GFRA1 and vector were purchased from Genomeditech and transduced into MFC cells using HitranasG reagent, and selected by incubation with puromycin.

### Reverse transcription and quantitative real-time PCR (qRT-PCR)

Trizol reagent (15,596,026, Ambin, CA, USA) was used for total RNA extraction. Next, total RNA was reversely transcribed into cDNA using a PrimeScript RT reagent Kit with gDNA Eraser (RR037A, Takara, Japan). Subsequent quantitative real-time PCR included TB green Premix EX TaqII (Takara #RR820A, Tokoyo, Japan) and a Light Cycler 96 System (Roche, Switzerland). Relative mRNA expression was calculated using the ΔΔCT method, with 18 s mRNA as internal reference and the highest ΔCT value as external reference. The primer sequences for RT-qPCR are listed in the supplementary table.

### Western blotting

Total cell proteins were prepared using IP lysate (P0013, Beyotime, China) mixed with protease and phosphatase inhibitors (HY-K0010 and HY-K0023, MCE, China). Cell lysates were centrifugated at 4℃ and their concentrations were determined using a BCA Protein Assay Kit (Pierce Biotechnology, USA). Next, the samples were subjected to separation through electrophoresis with 10–12% Tris SDS-PAGE gels and transferred to PVDF membranes. The membranes were incubated with 5% skimmed milk at room temperature and primary antibodies at 4℃ overnight. Next, secondary horseradish peroxidase (HRP) antibodies were applied at room temperature. Finally, proteins were visualized using ECL (WB012, Share-Bio, Shanghai) detection regent and a Bio-Rad system. The antibodies used were directed against GFRA1 (ab84106, Abcam, 1:1000), GDNF (ab18956, Abcam, 1:1000), RET (14,556, Cell Signaling Technology, 1:1000), β-actin (30101ES50, Yeasen, China, 1:5000), mTOR (2983, CST, 1:1000), p-mTOR (5536, CST, 1:1000), S6K (2708, CST, 1:1000), p-S6K (9204, CST, 1:1000), BECN1 (3495, CST, 1:1000), LC3 (12,741, CST, 1:1000), P62 (8025, CST, 1:1000), cleaved caspase-3 (9661, CST, 1:1000) and its corresponding HRP-conjugated antibodies (anti-mouse, 115–035-003 and anti-rabbit, 111–035-003, Jackson ImmunoResearch, 1:10,000 for both).

### Immunohistochemistry (IHC) assay

Patient and mouse tissues were embedded in paraffin and cut into 5 μm-thick sections for IHC analysis. Next, the sections were deparaffinized in xylene and rehydrated in an alcohol gradient (100–95-85–75%). After antigen retrieval using 10 mM citrate buffer (pH 6.0) at 95℃, the tissues were exposed to 0.3% hydrogen peroxide in methanol to eliminate endogenous peroxidases. After blocking with 10% BSA, the sections were incubated with primary antibodies overnight, followed by incubation with its corresponding secondary antibodies (HPR-conjugated antibodies, 1:300) at room temperature for 1 h. Next, target proteins were tagged using a DAB substrate kit (8059, Cell Signaling Technology, USA) and visualized under a microscope (Vector Laboratories, PK6200) after hematoxylin staining.

### Immunofluorescence assay

Tissue slides were processed according to the above protocols until antigen retrieval. Antigen blocking was conducted using 10% BSA after which incubation with primary antibodies was continued overnight. Next, secondary antibodies carrying green- or red-fluorescence were added to the slides at room temperature in the dark after which nuclei were stained with DAPI. Immunofluorescence was detected using confocal microscopy. Cells on chamber slides (81,201, ibidi, German) were fixed with 4% paraformaldehyde and permeabilized with 0.2% Triton X-100 (P0096, Beyotime, China). Next, the cells were incubated with primary antibodies and fluorescent secondary antibodies in the dark. *GFP-RFP-LC3* plasmids were transfected into AGS cells before treatment and next evaluated using confocal microscopy after DAPI staining. LysoTracker and LysoSensor were used to stain lysosomes.

### Cell viability assay

Cell viability was detected using a Cell Counting Kit 8 (CCK8, Dojindo, Japan). 2000 cells per well were seeded in 96 well plates with five repeated wells assigned for each group to evaluate proliferation or apoptosis of GC cells bearing genetic alternations. Proliferation was observed after incubation in medium supplemented 10% FBS and apoptosis after starvation induction in FBS-free medium. Absorption values at 450 nm were measured using a microplate reader after adding CCK8 reagent (1:10 with medium) for one hour daily. Cell viability values were recorded daily after the cells were seeded and treated as indicated. Proliferation and apoptosis curves were drawn based on the cell viability values. For apoptosis assays under conditioned culture conditions, supernatants from nc- or si*GDNF*- TAMs were collected and applied to GC cells cultured without FBS. 20 ng/ml rGDNF was used for rescue experiments.

### Flow cytometric apoptosis assay

Apoptosis was measured using a FITC-Annexin V and PI Apoptosis Kit (F6012, US Everbright, China) according to the supplier’s protocol. Gastric cancer cells (AGS and HGC-27) were cultured without FBS for 72 h, and then stained for measuring apoptotic rates. The stained cells were counted using flow cytometry and divided into groups based on their fluorescent signals.

### Colony formation under co-culture conditions

5 × 10^5^ TAMs were seeded into the upper ward of a Boyden chamber (0.4 μm, Millipore, USA), which was set in a six-well dish. Scramble and sh*GFRA1* transfected cells (1.5 × 10^5^) were placed in the bottom well and on the six-well dish. After one week, alive colonies on the dishes were fixed, stained and counted using representative fields. All assays were performed in triplicate and the results are shown as means ± SD.

### TdT-mediated dUTP nick-end labeling (TUNEL) assay

Human tissue sections were deparaffinized and hydrated as described above. Subsequently, the slides were processed using an In Situ Cell Death Detection Kit, Fluorescein (11,684,795,910, Roche, Switzerland). After nuclear staining with DAPI, the slides were photographed under a fluorescent microscope.

### Transmission electron microscopy (TEM)

AGS cells were fixed with 2% paraformaldehyde and 2% glutaraldehyde in 0.1 M phosphate buffer (pH 7.4), and then postfixed with 1% OsO4 for 2 h. After dehydration with increasing concentrations of alcohol (30, 50, 70, 90 and 100%), LR white resin (Sigma, 62,661) was infiltrated twice for 1 h, and the cells were embedded. The resulting solidified blocks were cut to 60 nm and stained with uranyl acetate and lead citrate. Ten regions were observed under a transmission electron microscope (Hitachi H-7600; Hitachi High-Tech Co., Tokyo, Japan) and representative photos were taken.

### Cytosolic Ca^2+^ level assay

Cytosolic Ca^2+^ levels were assessed using Fluo-4 AM (F14217, Invitrogen). AGS cells were loaded with 5 μM Ca^2+^ indicators in 1% BSA medium and then washed with PBS. The fluorescence intensity was measured at a specific wave length: Ex/Em = 494/516 nm. The next equation was applied to calculate free Ca^2+^ concentrations: [Ca^2+^]_free_ = Kd × (F − F_min_) / (F_max_ − F), where the Kd (ion dissociation constant) is 345 nM. F, F_max_ (maximal intensity) and F_min_ (minimal intensity) referred to the observed outputs in FBS-free medium after the addition of ionomycin (1 μM) and EGTA (10 mM). All assays were performed in triplicate and the results are shown as means ± SD.

### Bioinformatics

Next-generation sequence data were collected from 415 stomach adenocarcinoma (STAD) cancer samples in the TCGA to perform gene set enrichment analysis (GSEA). According to the cut-off value as median, the samples were divided into a low expression group (n = 208) and a high expression group (n = 207). Functional enrichment analyses were subsequently performed using GESA software. The significance threshold was set at *p* < 0.05. Detailed graphs labelled with a standardized enrichment score (NES) and *p* value were used to obtain gene set enrichment results. GEPIA, a website-based tool, was applied to differential gene expression analysis in major pathological stages of the 415 STAD samples from the TCGA, including profiling plotting. The TISIDB website was used to assess correlations between GFRA1 levels and macrophages. Spearman correlation plotting between GFRA1 and macrophages (Y axis) in STAD cancer (X axis) was performed.

### Statistical analysis

SPSS 26.0 and GraphPad 8 software were used for statistical analyses. Difference analyses were evaluated through pairwise comparisons of two-tailed Student's t-test or Welch’s t-test. A non-parametric Spearman correlation coefficient was used for correlation analysis. Overall survival curves were calculated using the Kaplan Meier method. Statistical significance was defined as *p* < 0.05.

## Results

### Pro-metastatic and anti-apoptotic effects of GFRA1 on GC cells

**Fig. 1:**
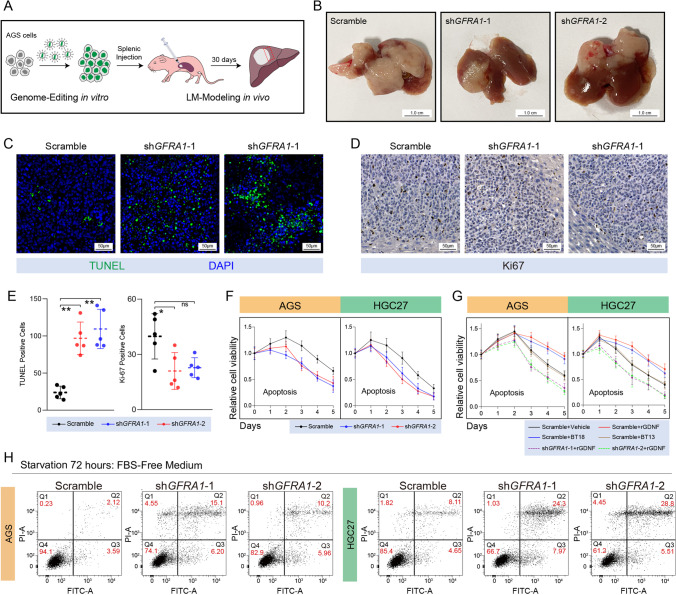
Oncogenic roles of *GFRA1 *in vivo and in vitro. (**A**) Protocol of *GFRA1*-silencing and liver-metastasis modelling. AGS cells were stably transfected with sh*GFRA1* and then injected into the spleen of nude mice for liver-metastasis formation. (**B**) Representative images of whole livers from LM mouse models. (**C**) Representative images of TUNEL (green) signals of above specimens derived from metastatic lesions. (**D**) Representative IHC images of Ki67-staining of above metastatic AGS tumors. (**E**) Histograms of TUNEL-positive cells and Ki67-positive cells of metastatic tumors as indicated. (**F**) Apoptotic curves of AGS (left panel) and HGC-27 (right panel) cells transfected with sh*GFRA1* or scramble plasmids, cultured in FBS-free medium over five days. (**G**) Apoptotic curves of AGS (left panel) and HGC-27 (right panel) cells in the presence of recombinant protein rGDNF (20 ng/ml), specific agonists BT18 (50 μM) or BT13 (50 μM), and pre-transfected with sh*GFRA1* or scramble plasmids. (**H**) Representative flow cytometric apoptosis assays of AGS (left panel) and HGC-27 (right panel) cells transfected with above plasmids in FBS-free medium. Scale bar: respectively labelled in every module, “ns” not significant, *p < 0.05, **p < 0.01

A genome-wide DNA methylation sequencing study revealed that the methylation level of the GFRA1 promoter region was significantly reduced in GC patients with liver metastasis, suggesting that the transcriptional upregulation of *GFRA1* may be closely related to the formation of GC liver metastases. Therefore, we examined the mRNA levels of the *GFRA* family and their ligand family members in normal gastric mucosal cells and in six GC cell lines. Additionally, we selected the highly-expressing AGS and HGC-27 cell lines to construct stable *GFRA1*-knockdown GC cells (Fig. S1A). To construct a liver metastasis model in vivo, gene-edited AGS cells were injected into the spleen of nude mice (Fig. 1A). We found that *GFRA1* downregulation significantly inhibited the colonization and formation of metastatic GC niches in the liver (Fig. 1B). Additionally, we collected metastatic tissues for pathological examination and found that *GFRA1* silencing increased the TUNEL-positive signalling of GC cells in metastatic lesions and slightly decreased Ki67 expression of these cells (Fig. 1C-E). Using a cell viability assay we found that inhibition of *GFRA1* had little impact on the proliferative abilities of AGS and HGC-27 cells, while it moderately weakened their anti-apoptotic ability in vitro (Fig. 1F, S1B). Specific agonists and recombinant protein were used to validate the oncogenic mode of action of the GDNF-GFRA1 axis. The specific agonists of GFRA1, BT18 and rGDNF, markedly reduced the apoptosis of GC cells under starvation, but had little effect on their proliferative activity (Fig. 1G, S1B). The protective effects of BT18 and rGDNF on GC cells could be fully eliminated by *GFRA1* silencing (Fig. 1G). However, BT13, as agonist of the GFRA3 receptor, barely affected their anti-apoptotic ability (Fig. 1G). Additional apoptotic assays confirmed that *GFRA1* deregulation increased cell apoptosis triggered by starvation (Fig. 1H). Using a wound healing assay, we found that the *GFRA1* levels were not associated with the migrative ability of GC cells (Fig. S1C-D). Together, these data indicate that GFRA1 promotes the formation of liver GC metastatic niches. The in vitro data indicate that GFRA1 mainly helps cells to resist apoptosis, and that this function depends on GFRA1 activation by its exogenous ligand GDNF.

### Expression profiles of GDNF and GFRA1 in invasive margins

**Fig. 2:**
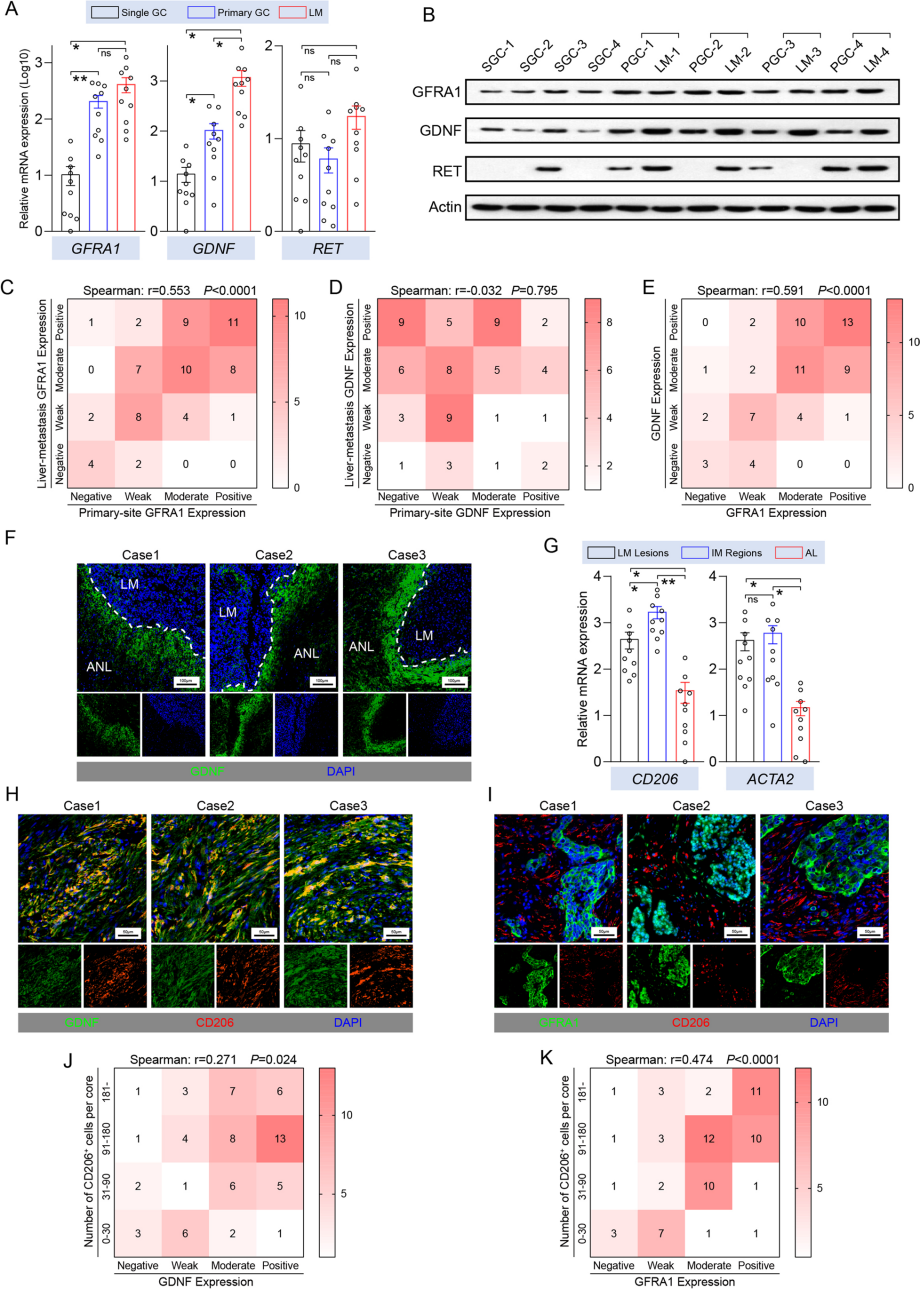
Expression profiles of GDNF and GFRA1 in metastatic GC specimens. (**A**) Relative *GFRA1*, *GDNF* and *RET* mRNA levels in tumor tissues from single primary and metastatic GC patients (n = 10, respectively). (**B**) Protein levels of *GFRA1*, *GDNF* and *RET* in tumor tissues from single primary and metastatic GC patients. SGC: single gastric cancer, PGC: primary GC and LM: liver metastasis. (**C**-**E**) Correlation analysis of protein levels in a tumor microarray by IHC scores (n = 69, in total). Heatmap of GFRA1 levels between primary gastric and metastatic liver lesion shown in C, heatmap of GDNF levels between primary gastric and metastatic liver lesion shown in C, and correlation between GFRA1 and GDNF levels in metastatic lesions shown in E. (**F**) Representative immunofluorescent images of GDNF (green) in metastatic liver lesions from three GC cases. ANL: adjacent normal liver. (**G**) Relative *CD206* and *ACTA2* mRNA levels in tissues from different regions (n = 10), which encode CD206 and SMA, respectively. IM: invasive margin tissues and AL: adjacent liver tissues. (**H**) Double-staining of GDNF and CD206 in metastatic lesions from three GC cases. (I) Double-staining of GFRA1 and CD206 in metastatic lesions from three GC cases. (**J**-**K**) Correlation analysis of protein levels ina tumor microarray by IHC scores (n = 69, in total). Heatmap of GDNF levels and CD206 positive cells in metastatic tissues shown in K, and heatmap of GFRA1 levels and CD206 positive cells shown in L. Scale bar: respectively labelled in every module, “ns” not significant, *p < 0.05, **p < 0.01

Our cell models indicated that the GDNF-GFRA1 axis is involved in the regulation of GC liver metastasis. To verify that such a phenomenon also occurs in clinical cases, we examined the expression levels and distribution patterns of GDNF, GFRA1 and RET proteins in GC liver metastatic tissues. We found that the GFRA1 expression levels in the primary tumor tissues and in the liver metastases of metastatic GC patients were higher than those of patients with primary tumors only, but no significant differences between the primary lesions and metastases were observed. The GDNF expression pattern was slightly different, and its expression in liver metastases was significantly higher than that in primary tumors of metastatic GC patients and in primary tumor only cases (Fig. 2A-B). Tissue microarrays containing samples from 69 GC patients with liver metastases confirmed this finding. Moreover, we observed a strong positive correlation between the expression levels of GFRA1 in primary and metastatic niches in patients with metastatic GC, whereas no correlation was observed between GDNF expression levels in the two types of niches, with a higher expression in metastatic niches (Fig. 2C-D). In addition, we found that the GFRA1 protein was mainly expressed on the surfaces of the tumor cells, and that GDNF was mainly expressed in stromal cells (Fig. S1A-B). We also observed a strong positive correlation between the expression levels of the two proteins in liver metastases (Fig. 2E). Unexpectedly, as a classical co-receptor of the GFRA1 protein, the RET protein and its coding gene were basically not expressed in the multiple GC cell lines tested, and their expression levels were not associated with GC liver metastasis, suggesting that GFRA1 may promote GC liver metastasis through a nonclassical RET-independent pathway (Fig. 2A, S1A).

We also found that there was a significant enrichment of the GDNF protein at the invasive margins of the GC liver metastatic niches (Fig. 2F), which are the frontiers for the interaction between tumor cells and the metastatic microenvironment and harbour abundant stromal cells, such as TAMs and cancer-associated fibroblasts (CAFs). This notion was underscored by detecting the expression levels of TAM and CAF markers after extracting RNA from normal liver tissues, tumor tissues and invasive margin tissues of liver metastatic niches (Fig. 2G). In addition, a fluorescence-based analysis confirmed a high degree of co-localization of CD206^+^ TAMs and the GDNF protein (Fig. 2H). Also, many CD206^+^ TAMs were found to be present next to GC cells with a high GFRA1 expression (Fig. 2I). All previous findings were confirmed via correlation analysis by IHC scoring using a tissue microarray of GC liver metastases (Fig. 2J-K). A bioinformatic analysis using the Cancer Genome Atlas (TCGA) database at the TISIDB website also revealed that GFRA1 expression levels in GC tissues are closely related to macrophage infiltration (Fig. S2C). These results indicate that the invasive margins of GC liver metastatic niches exhibit abundant TAM infiltration. Additionally, they suggest that the GFRA1 protein involved in the formation of liver metastases needs to be activated by its ligand GDNF, which is derived from infiltrating TAMs in the metastatic microenvironment.

### The oncogenic function of GFRA1 requires TAM-derived GDNF in vitro and in vivo

**Fig. 3:**
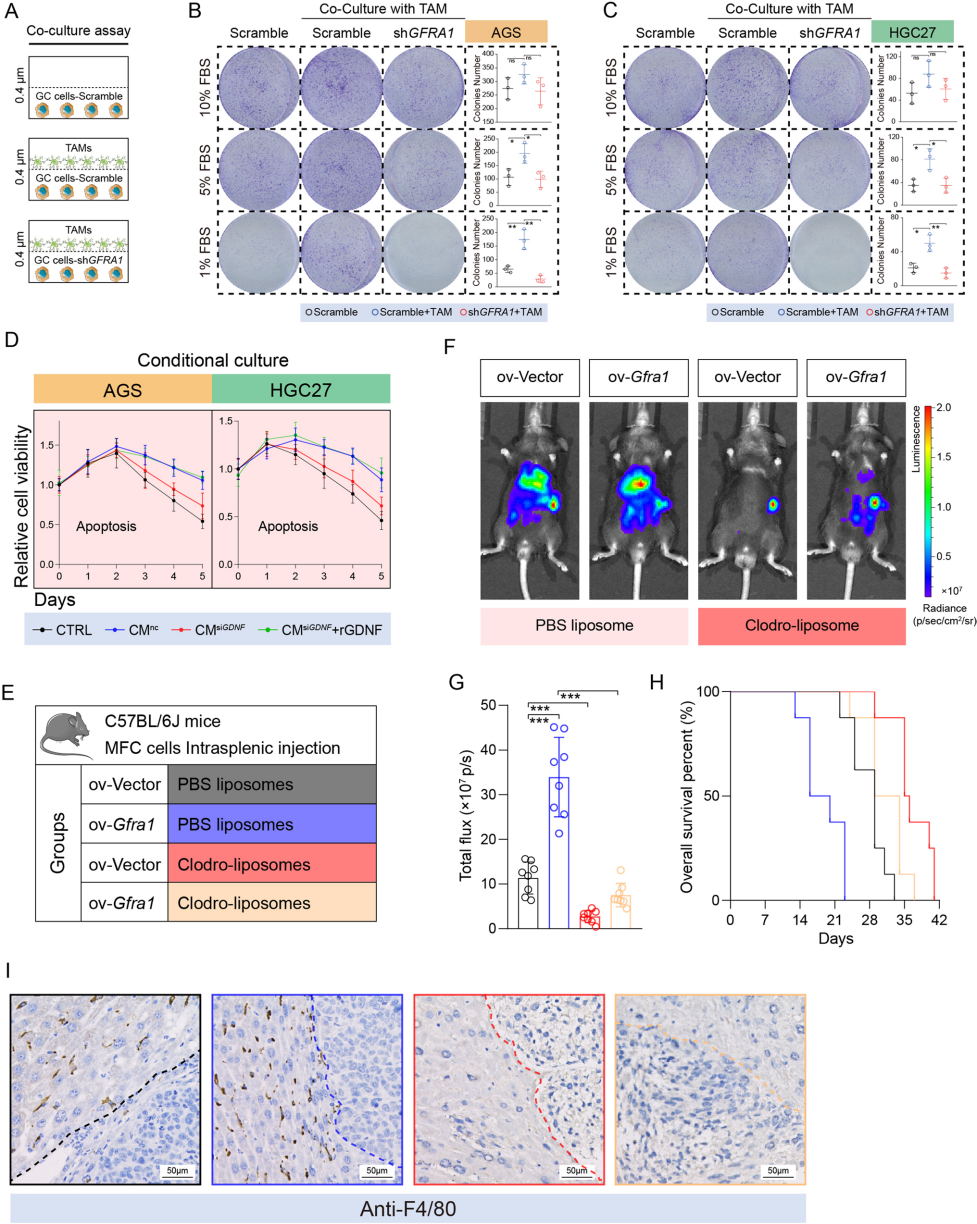
TMA-endowed malignancy of GFRA1-positive cells in vitro and in vivo. (**A**) Diagram of co-culture assay between tumor associated macrophages (TAMs) and GC cells. (**B**-**C**) Representative images and histograms of co-culture colony formation under gradient concentrations of fetal bovine serum (n = 3, respectively). (**D**) Apoptotic curves of AGS (left panel) and HGC-27 cells (right panel) cultured in conditioned medium (CM) collected from nc- or si*GDNF*- TAMs. (**E**) Mice grouping information (n = 8 for each group). Ov: overexpressing and Clodro: Clodronate. (**F**) Representative images of luminescence emitted by LM-model mice 4 weeks after intrasplenic injection. (**G**) Histogram showing the luminescence intensity of model mice (n = 8, respectively). (**H**) Survival curves of four groups of indicated mice (n = 8, respectively). (**I**) Representative IHC images showing the F4/80 positive cells in metastatic lesions from model mice. Scale bar: 50 μm. “ns” not significant, *p < 0.05, **p < 0.01, ***p 0.001

We used the human monocytic cell line THP1 to induce in vitro differentiation to obtain TAMs, after which the cells and their supernatants were used for co-culture and conditional culture experiments, respectively (Fig. S3A). We found that the *GDNF* gene was transcriptionally upregulated in TAMs, compared with Mφ macrophages (Fig. S3B). Subsequent co-culture of TAMs with GC cells revealed that the TAMs acted on GFRA1 expressed by the GC cells through secreted proteins to enhance their growing ability under poor nutritional conditions (Fig. 3A-C). In addition, supernatants of cultured nc- and si*GDNF*-TAM cells were collected for the conditional culture of GC cells, after which an apoptotic curve was drawn. We found that GDNF expression silencing in TAMs caused them to lose the ability to promote GC cells to resist apoptosis, which could subsequently be restored by adding exogenous recombinant GDNF protein (Fig. 3D).

C57 mice were intraperitoneally injected with disodium chlorophosphate liposomes 2 weeks before modelling to eliminate macrophages, after which mouse-derived GC MCF cells overexpressing GFRA1 were injected into the spleen to construct a liver metastasis model (Fig. 3E, S3C). We found that GC liver metastasis was significantly dependent on GFRA1 overexpression. When macrophages were eliminated from the mice, GFRA1-dependent metastatic focus formation was significantly inhibited (Fig. 3F-G). The survival of mice with GC liver metastases was also significantly prolonged after the elimination of macrophages (Fig. 3H). IHC analysis of the livers of the mice confirmed that the macrophages were indeed be eliminated by disodium chlorophosphate liposomes. In conclusion, these results suggest that GFRA1, which is highly expressed in GC cells, relies on TAM-derived GDNF to promote tumor cells to resist apoptosis and to form liver metastases.

### GFRA1 controls cellular autophagy flux by modulating fusion with lysosomes

**Fig. 4:**
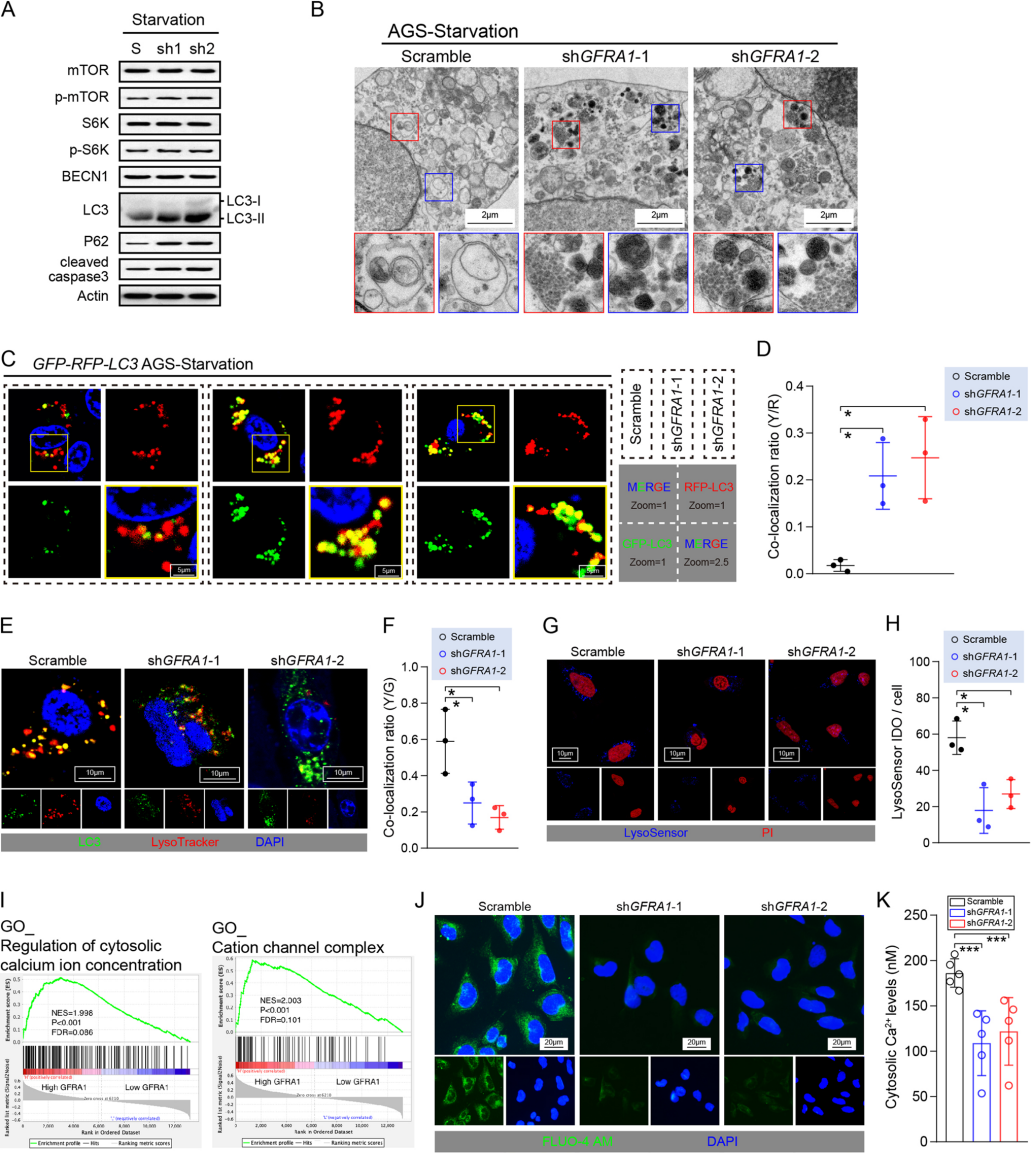
The GDNF-GFRA1 axis modulates the cellular autophagy-lysosome system. (**A**) Intracellular levels of mTOR, S6K, and the autophagy-related proteins BECN1, LC3, P62 and cleaved caspase 3 under starvation. (**B**) Representative images of transmission electron microscopy showing the levels of autophagosomes in scramble and sh*GFRA1* cells. (**C**) Representative images of AGS cells transfected with GFP-RFP-LC3 plasmids. Corresponding signals are labelled in the right lower corner. (**D**) Co-localization ratios of yellow vs. red (Y/R) puncta in the GFP-RFP-LC3 fluorescent models (n = 3, respectively). (**E**) Double staining for LC3 (green) and LysoTracker (red) in AGS cells in which *GFRA1* is silenced. (**F**) Co-localization ratios of yellow vs. green (Y/G) puncta shown in a histogram (n = 3). (**G**) LysoSensor assay in AGS cells in which *GFRA1* is silenced. Positive LysoSensor signal shown in blue and nuclear dye PI shown in red. (**H**) Histogram showing the mean fluorescent intensity of single cells using a LysoSensor assay (n = 3). (**I**) GSEA results of GC specimens from the TCGA database, grouped by *GFRA1* expression levels. (**J**) Representative images of a FLUO-4 AM assay in scramble and sh*GFRA1* AGS cells, reflecting intracellular Ca^2+^ concentrations. (**K**) Histogram showing cytosolic Ca^2+^ levels calculated by FLUO-4 AM assay. Scale bar: respectively labelled in every module, “ns” not significant, *p < 0.05, ***p < 0.005

Next, we examined the level of autophagic flux in AGS cells under starvation and found that *GFRA1* silencing significantly increased the level of light chain 3 (LC3)-II protein and the conversion rate of LC3-II/I. Additionally, we observed p62 protein accumulation (Fig. 4A), which suggests that *GFRA1* inhibition may block the autophagic flux in cells. The phosphorylation levels of the mammalian target of rapamycin (mTOR) protein and its downstream S6K protein were not affected by *GFRA1* silencing, and the BECN1 protein levels were unchanged. Therefore, we conclude that the inhibition of GFRA1 signalling affects autophagic flux activation and autophagosome production (Fig. 4A). Moreover, we found that blockage of the autophagic flux was accompanied by an increase in cleaved caspase3 levels, which is a marker of apoptosis (Fig. 4A). After knocking down GFRA1, the phenomenon of autophagic flux blockage and autophagosome accumulation in cells could directly be observed using transmission electron microscopy (TEM). Many autophagosomes did not fuse with lysosomes and were degraded by the latter (Fig. 4B). In addition, we used a GFP-RFP-LC3 double fluorescent plasmid to label autophagosomes in AGS cells and found that when GFRA1 was knocked down, a high degree of a yellow fluorescence signal appeared in the cells. The unstable green fluorescent protein (GFP) dissipated rapidly in lysosomes, suggesting that the autophagosomes produced under starvation conditions were not successfully degraded (Fig. 4C-D). Further labelling of lysosomes revealed that interfering with GFRA1 may render the autophagosomes produced in the cells unable to fuse with lysosomes, thus allowing them to enter the degradation process (Fig. 4E-F). Such degradation mainly occurs because when GFRA1 is inhibited, lysosomes in the normal pH range inside of the cells are significantly reduced, and the signal of the LysoSensor probe that can label them is significantly attenuated (Fig. 4G-H). It has been reported that GDNF-GFRA1 signalling may be involved in regulating the intracellular calcium concentration, which is a key signal transduction molecule in the regulation of lysosome formation and function. Here, we performed a gene set enrichment analysis (GSEA) after dividing the GC sequencing data in the TCGA database into two groups (according to GFRA1 expression), and found that GFRA1 was indeed closely related to calcium signalling in GC (Fig. 4I). Intracellular calcium concentration detection using a FLUO-4 AM probe confirmed that GFRA1 levels are closely related to calcium signalling, which may also be the mechanism by which the GDNF-GFRA1 signalling axis controls the level of autophagic flux in GC cells (Fig. 4J-K).

### Autophagy mediates the oncogenic function of GFRA1 and is accessible to be targeted

**Fig. 5:**
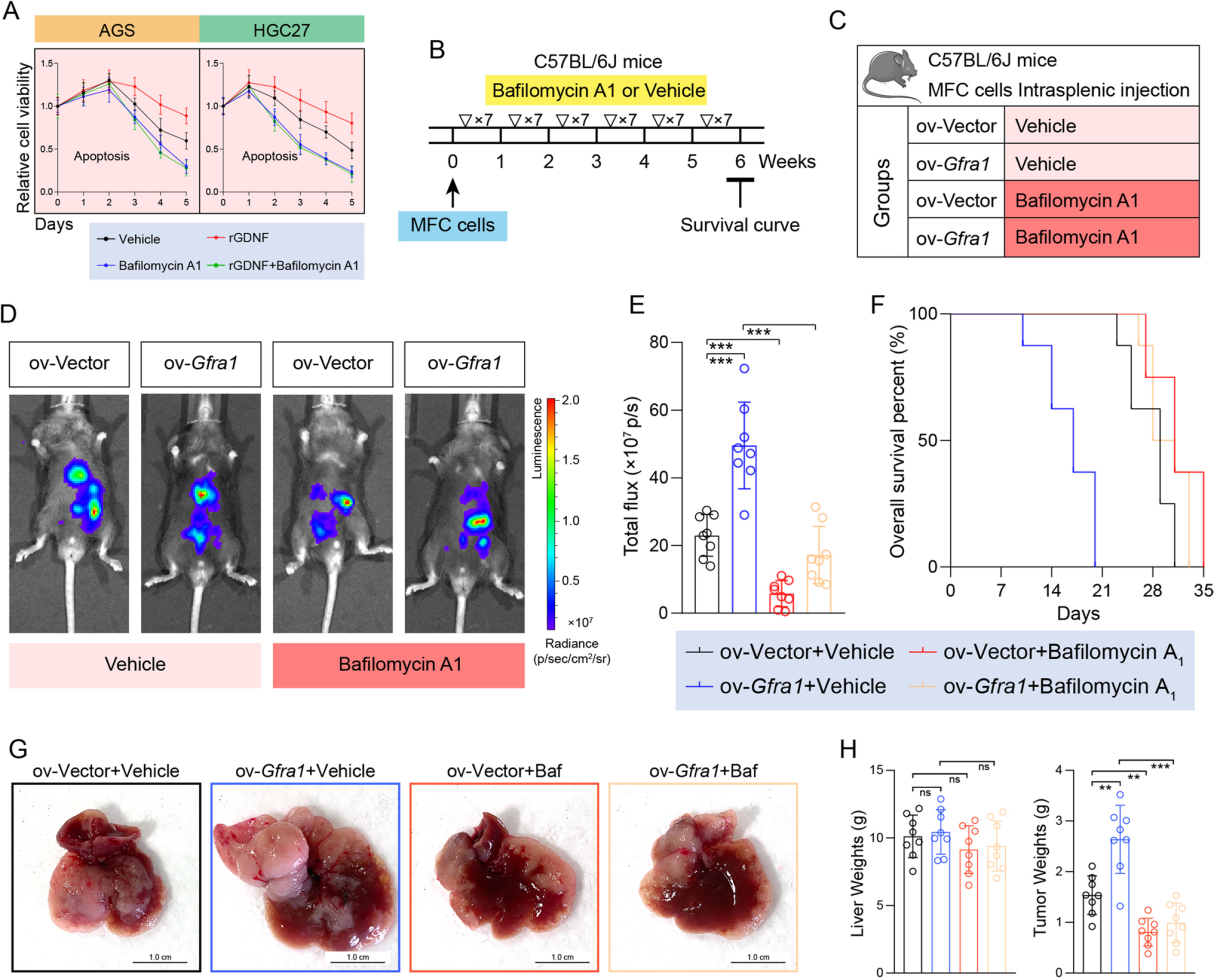
GFRA1-dependent liver metastasis is inhibited by autophagy-targeted treatment. (**A**) Apoptotic curves of AGS cells (left panel) and HGC-27 cells (right) incubated with 20 ng/ml rGDNF or 100 nM autophagy inhibitor bafilomycin A_1_. (**B**) Flow diagram of LM modelling and inhibitor treatment of mice. 1 mg/kg bafilomycin A_1_ was intraperitoneally injected per day till six weeks. (**C**) Mice grouping information (n = 8 for each group). (**D**) Representative images of luminescence emitted by LM-model mice, 4 weeks after intrasplenic injection. (**E**) Histogram showing the luminescence intensity of model mice (n = 8, respectively). (**F**) Survival curves of these four groups of mice (n = 8, respectively). (**G**) Representative images of gross livers from LM-bearing mice. (**H**) Histogram showing the weights of livers and metastatic hepatic tissues, respectively (n = 8). Scale bar: respectively labelled in every module, “ns” not significant, **p < 0.01, ***p < 0.001

Our initial preclinical experiment involved an autophagic inhibitor to verify the above-described findings. We found that exogenous recombinant GDNF protein could significantly increase the anti-apoptotic abilities of AGS and HGC-27 cells, whereas such effects could be completely abolished via the autophagy inhibitor bafilomycin A1 (Fig. 5A). Bafilomycin A_1_ (1 mg/kg) was used to treat mice with liver metastases, and the inhibitor was injected intraperitoneally daily (Fig. 5B-C). Consistently, we found that the niche scope of GFRA1-dependent liver metastases could be significantly inhibited by bafilomycin A_1_, which was accompanied by a prolonged survival of the mice (Fig. 5D-F). The growth of metastatic lesions was also inhibited by bafilomycin A_1_, along with decreased tumor weights (Fig. 5G-H). These results not only confirm that GFRA1 may play a pro-metastatic role by regulating the autophagic flux, but also suggest that autophagic flux inhibitors may be used for the treatment of GC patients with liver metastases.

### GFRA1 expression levels correlate with a poor prognosis of GC patients

**Fig. 6:**
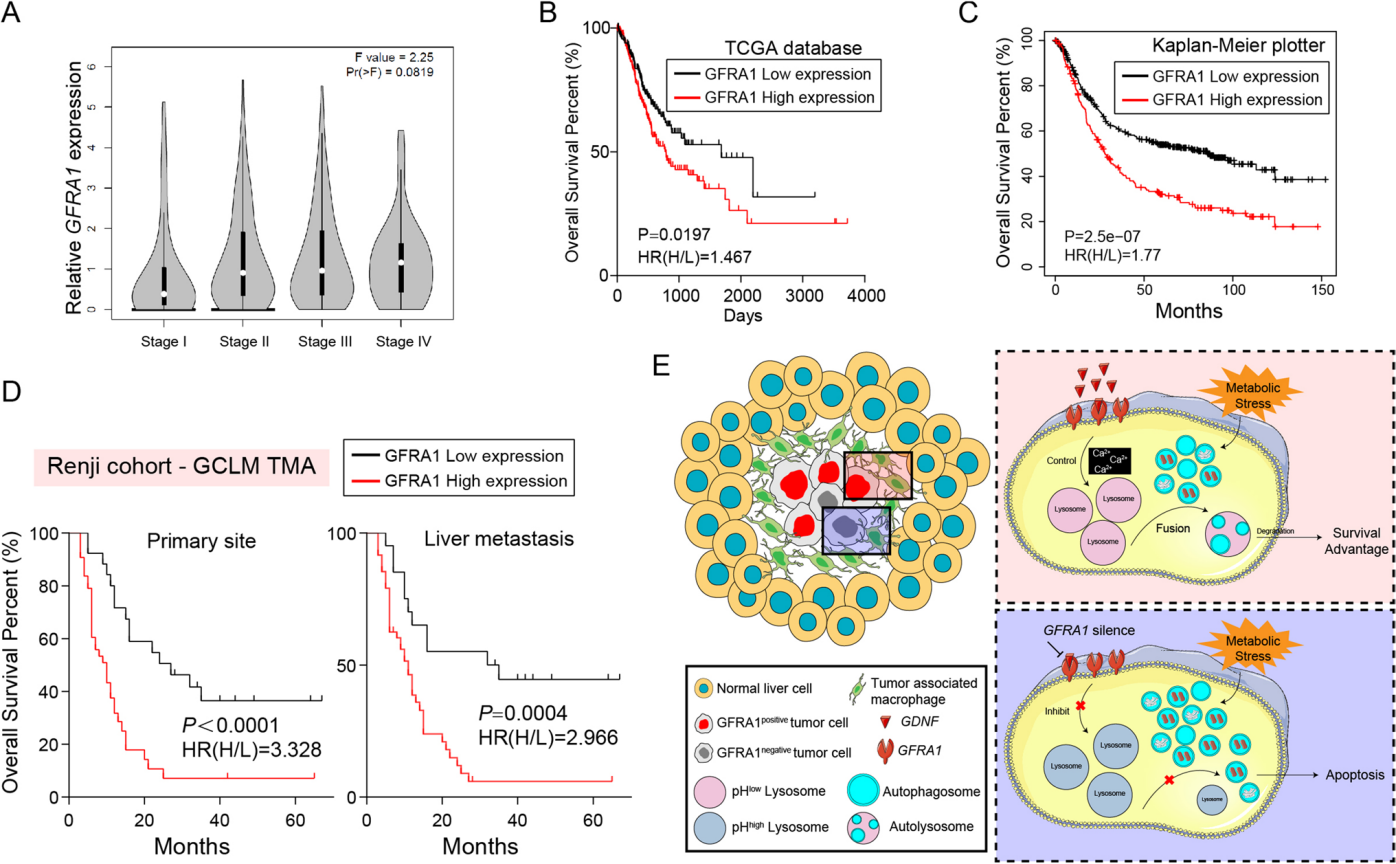
Expression levels of GFRA1 correlate with a poor prognosis of GC patents. (**A**) Increasing *GFRA1* levels along with TNM stages of GC patients in the TCGA database. (**B**-**C**) Survival curves based on the TCGA database (**B**) and the Kaplan–Meier Plotter website (**C**), analysed using the Kaplan–Meier method. The cut-off value was defined as median. HR(H/L): Hazard ratio of high vs. low expression. (**D**) Kaplan–Meier plots based on our liver-metastatic tumor microarray (n = 69), split by IHC scores. GFRA1 levels of primary lesions are shown in the left panel, and those in metastatic lesions in the right panel. (**E**) Schematic diagram of the anti-apoptotic mechanism in invasive margins through GFRA1-regulated autophagosome-lysosome fusion under metabolic stress

The GC data in the TCGA database show that the expression of GFRA1 gradually increases with tumor-node-metastasis (TNM) stage, with stage IV metastatic GC being highest, suggesting that its expression is closely related to the malignant progression of GC (Fig. 6A). The TCGA database and Kaplan–Meier plotter website also confirm that GC patients with a high GFRA1 expression have a poor prognosis (Fig. 6B-C). A tissue microarray containing samples from 69 GC patients with liver metastases showed that the GFRA1 protein level was also closely related to patient prognosis, and that high GFRA1 expression levels in both primary lesions and metastases indicate a poor prognosis (Fig. 6D). Overall, our results from clinical samples confirm that GFRA1 expression is closely related to patient prognosis and is significantly increased in metastatic stage IV GC, whereas for patients with metastatic GC, a high GFRA1 expression also indicates a poor prognosis.

## Discussion

At present, only a few studies have been reported on the mechanism of GC liver metastasis, mainly due to the difficulty to obtain clinical liver metastasis samples and a lack of stable animal models [6, 30]. Nevertheless, liver metastasis may seriously affect patient survival and prognosis and represents an important cause of GC patient death. As such, it has become a difficult problem in clinical diagnosis and treatment [8, 31]. Here, we performed a series of cell biological and oncology-related experiments to identify the mechanism underlying GC liver metastasis and identified GFRA1 as being a reliable biomarker and potential therapeutic target for GC patients with liver metastasis.

Adequate clinical samples and resources from public databases were included in this study to validate the aforementioned conclusions. Interestingly, we found individual differences in the expression of GFRA1 protein, i.e., the expression level of GFRA1 protein in tumor tissues of patients with metastatic GC was found to be significantly higher than that in tumor tissues of patients with only primary GC, whereas there was no significant difference between primary and metastatic niches or between GC tissues and adjacent normal mucosal tissues. In contrast, differences in GDNF protein expression were found between microenvironments, i.e., a specific high expression of GDNF was found in the invasive margins of liver metastases [32, 33]. This phenomenon suggests that for GC patients with a high GFRA1 expression in their tumor cells, the likelihood of liver metastasis is greater, but synergistic changes in the target organs are required. Specifically, a pre-metastatic niche is required, with infiltration of TAMs together with the accumulation of GDNF protein [34, 35]. Liver metastasis of GC depends on the malignant evolution of GC cells together with a "co-evolution" of the microenvironments of the target organ. Intervention at any link may facilitate the treatment of patients with metastatic GC [36–38].

It has been proposed that the formation of tumor metastases can occur at early developmental stages of the primary tumor. Therefore, the actual benefits of simply studying the processes of tumor cell invasion and migration in primary niches are often limited [39]. Here, we mainly focused on the processes of colonization and survival of GC cells in the metastatic niche, shedding light on the mechanism driving distant metastasis of tumors from another perspective [40, 41]. As such, our data may provide a new and reliable strategy for the treatment of patients with refractory GC. The expression level of GFRA1 can be used as a biomarker to assess the possibility of liver metastasis recurrence in patients with advanced GC. In addition, suitable patients may be selected based on their GFRA1 expression level, and such patients may benefit from targeted GDNF-GFRA1 axis treatment [42, 43].

Unfortunately, no reliable specific GFRA1 inhibitor is available, and we found that its classical co-receptor RET protein does not play a role in this process. Therefore, suitable drugs that can be used for clinical translation in patients with metastatic GC are currently lacking [44–46]. Thus, we further analysed the noncanonical mechanism driven by GFRA1 during GC liver metastasis and found that control over the autophagic activity of GC cells is a main mechanism [21, 28]. By using autophagy inhibitors, we found that the tumorigenic effect of GFRA1 could be significantly weakened, and that GFRA1 had a high inhibitory efficiency for GC liver metastasis in mice. As a result, we propose that for patients with GFRA1-dependent liver metastasis, personalized therapy targeting cellular autophagy may have clinical translational prospects. Many studies have proposed that virus-mediated targeted therapy can be used as an important strategy for clinical translation. For this, the *GFRA1* gene may also be a suitable target, but this requires additional studies to further explore this possibility [47, 48].

In general, we conclude that during GC liver metastasis tumor cells express GFRA1 and are highly dependent on GDNF secreted by TAMs in the microenvironment at the invasive margin. Noncanonical signal transduction downstream of the GDNF-GFRA1 axis is achieved by affecting the concentration of intracellular second messengers (calcium ions), which in turn regulate lysosomal function, maintain the level of autophagic flux under metabolic stress, help tumor cells to survive and colonize in the metastatic microenvironment of the liver and, ultimately, promote the occurrence and development of GC liver metastases [49, 50]. Our findings not only provide new insights in the mechanism of GC liver metastasis, but also indicate a new strategy for personalized treatment and disease management of patients with metastatic GC.

## Supplementary Information

Below is the link to the electronic supplementary material.

Supplementary file1 (JPG 1046 kb)

Supplementary file2 (JPG 1929 kb)

Supplementary file3 (JPG 314 kb)

Supplementary file4 (DOCX 14 kb)

## Data Availability

The data and material supporting the conclusion of this study have been included within the article. Survival analysis of GFRA1 levels is based on the data from TCGA database and Kaplan-Meier Plotter website (http://kmplot.com/analysis/).
