# Supplementary figures and images for: Tumor-associated macrophage-derived GDNF promotes gastric cancer liver metastasis via a GFRA1-modulated autophagy flux
Source: Cell Oncol (Dordr). 2023 Feb 20;46(2):315–30. doi: 10.1007/s13402-022-00751-z (PMC10060314; doi:10.1007/s13402-022-00751-z)

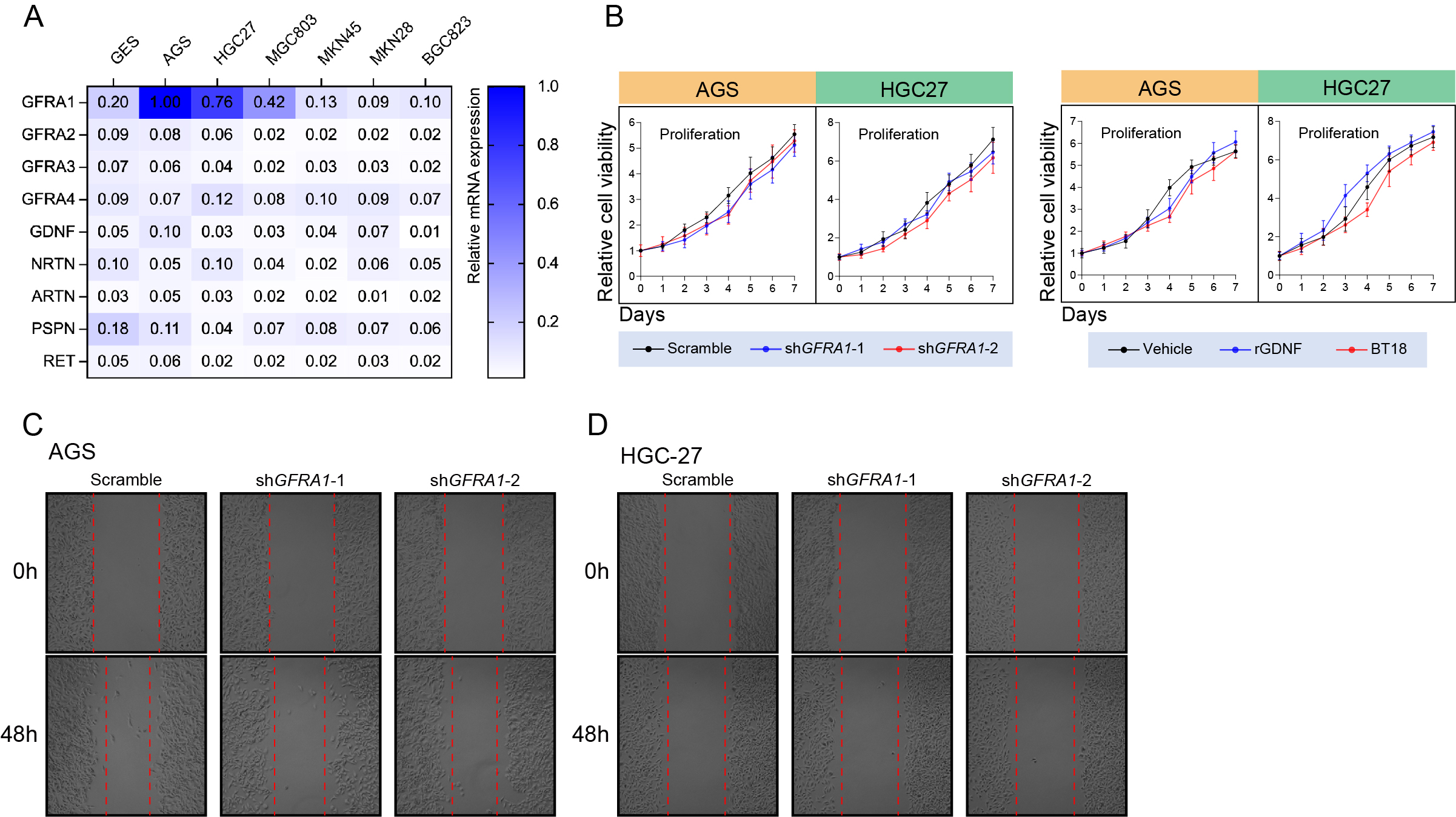

Supplement: Supplementary file 1 — Supplementary file1 (JPG 1046 kb) [file 13402_2022_751_MOESM1_ESM.jpg]

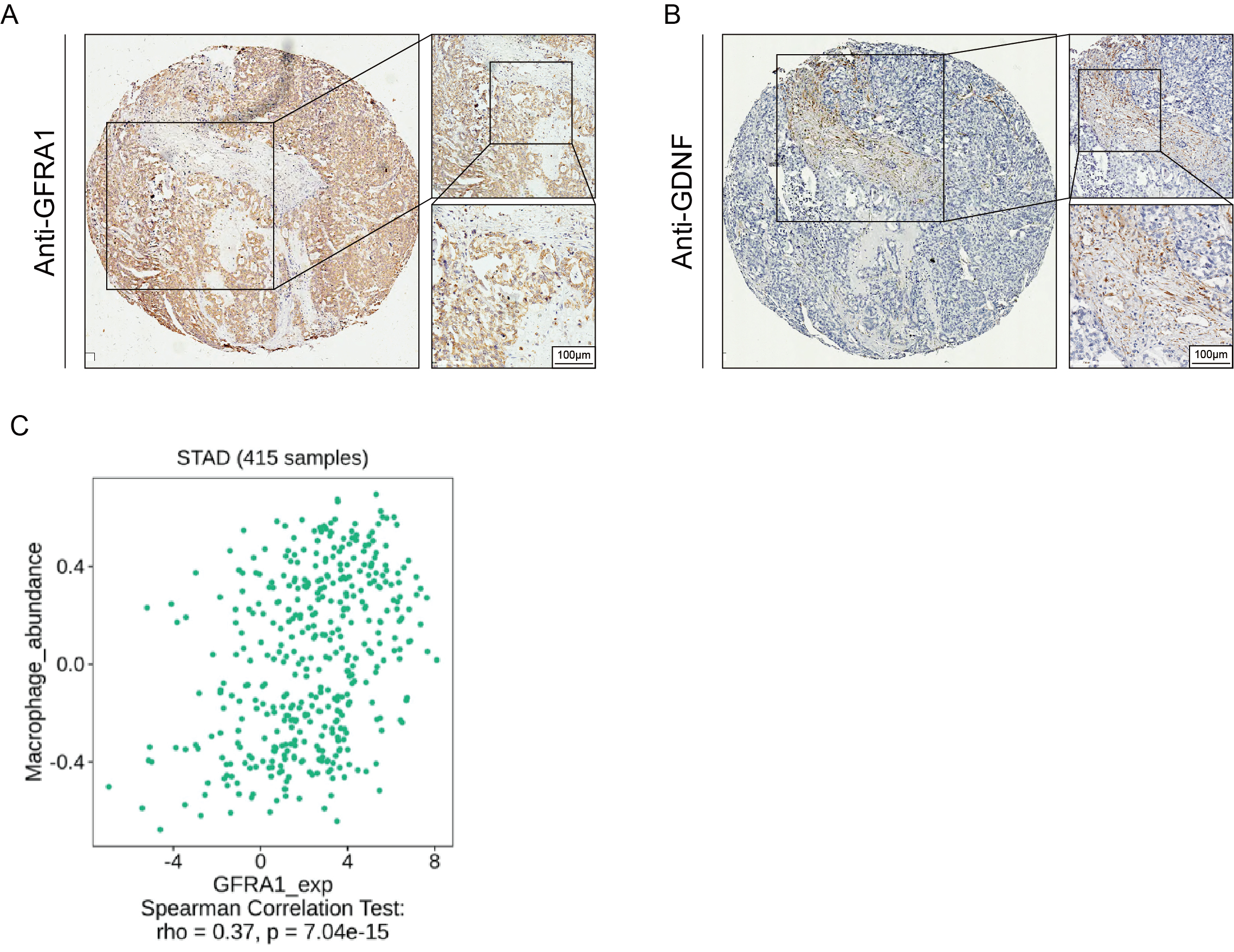

Supplement: Supplementary file 2 — Supplementary file2 (JPG 1929 kb) [file 13402_2022_751_MOESM2_ESM.jpg]

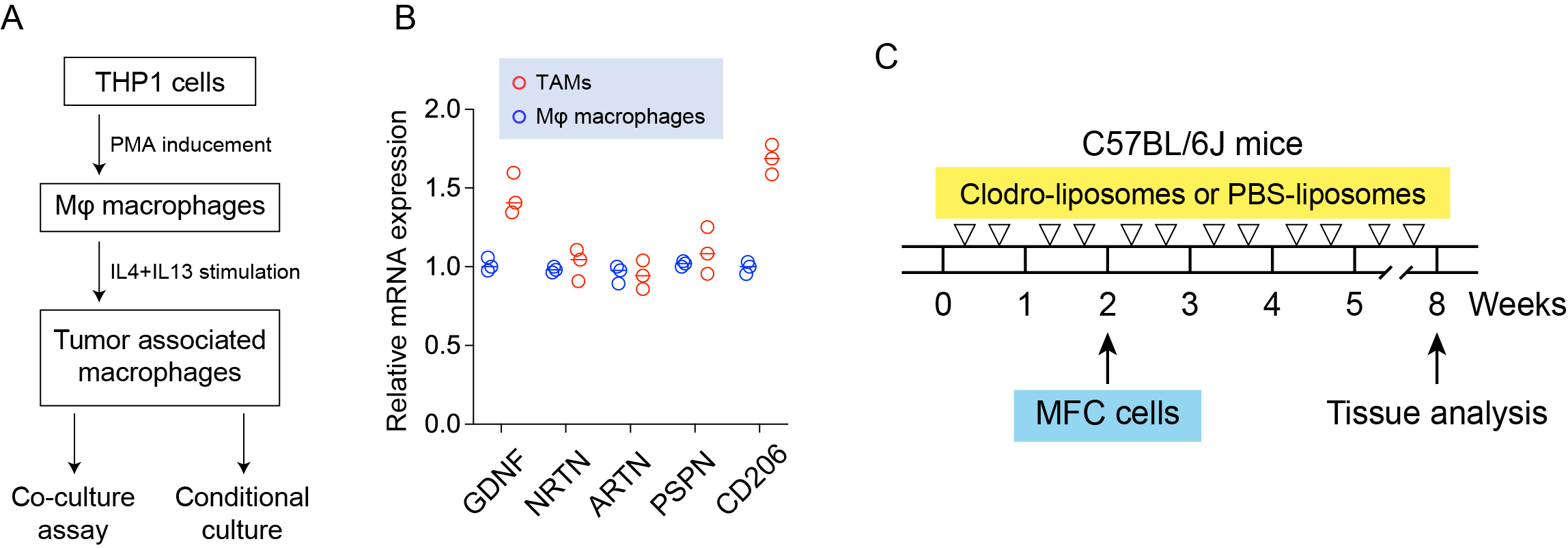

Supplement: Supplementary file 3 — Supplementary file3 (JPG 314 kb) [file 13402_2022_751_MOESM3_ESM.jpg]
