# Supplementary material for: Tumor-associated macrophage-derived GDNF promotes gastric cancer liver metastasis via a GFRA1-modulated autophagy flux
Source: Cell Oncol (Dordr). 2023 Feb 20;46(2):315–30. doi: 10.1007/s13402-022-00751-z (PMC10060314; doi:10.1007/s13402-022-00751-z)
Supplement: Supplementary file 4 — Supplementary file4 (DOCX 14 kb) [file 13402_2022_751_MOESM4_ESM.docx]

**Supplementary Figure Legends**

**Supplementary Figure 1**.

(A) Relative mRNA levels of *GFRA1* family members, *GDNF* family members and co-receptor *RET* in one normal epithelial cell and six cancer cell lines of stomach. (B) Proliferative curves of AGS and HGC-27 cells transfected with sh*GFRA1* plasmids (left panels). Proliferative curves of cells incubated with recombinant protein rGDNF and specific agonist BT18 (right panels). (C-D) Representative images of wound healing conducted with AGS and HGC-27 cells.

**Supplementary Figure 2**.

(A-B) Representative IHC images of GFRA1 and GDNF in LM tumour microarray. Scale bar: 100μm. (C) Correlation analysis between GFRA1 expression levels and macrophage abundance, calculated by website.

**Supplementary Figure 3**.

(A) Inducing protocol of tumor associated macrophages from THP-1 cell line. (B) Relative mRNA expression levels of *GDNF* family and *CD206* genes, red indicates TAMs and blue indicates Mϕ macrophages. (C) Flow diagram of macrophage deletion and LM modeling *in vivo*. 1.4mg/20g Clodronate liposomes and equivalent PBS liposomes were intraperitoneally injected twice a week till eight weeks.
